# Self-Reported Awareness and Adherence to Recommendations for Dietary Fat Amongst Australian Adults Aged 18–45 Years: Potential Relevance to Cardiovascular Health

**DOI:** 10.3390/nu18193236

**Published:** 2026-09-30

**Authors:** Elmira Karimi, Barbara Brayner, Lewan Parker, Jenna McVicar, Michelle A. Keske, Sian A. O’Gorman, Kimberley L. Way, Gunveen Kaur

**Affiliations:** 1 Deakin Institute for Physical Activity and Nutrition, School of Exercise and Nutrition Sciences, Deakin University, Geelong, VIC 3220, Australia; e.karimi@deakin.edu.au (E.K.); barbara.vitorinoalencarbrayner@deakin.edu.au (B.B.); lewan.parker@deakin.edu.au (L.P.); jenna.mcvicar@monash.edu (J.M.); michelle.keske@deakin.edu.au (M.A.K.); sogorma@deakin.edu.au (S.A.O.); kim.way@deakin.edu.au (K.L.W.); 2 Baker Heart and Diabetes Institute, Melbourne, VIC 3004, Australia; 3 Exercise Physiology and Cardiovascular Health Lab, Division of Cardiac Prevention and Rehabilitation, University of Ottawa Heart Institute, Ottawa, ON K1Y 4W7, Canada

**Keywords:** dietary fats, nutrition, dietary guidelines, prevention, cardiovascular disease

## Abstract

Background: In Australia, the National Health and Medical Research Council provides Nutrient Reference Values (NRVs) and the Australian Dietary Guidelines (ADG) to support public understanding of dietary fat intake and its role in cardiovascular as well as overall health. However, little is known about how effectively these recommendations reach and influence the public. This study examined self-reported awareness and beliefs related to dietary fats, with a particular focus on adherence to NRVs and ADG. Methods: A cross-sectional online survey was conducted using Qualtrics among Australian adults aged 18–45 years. Data were analysed using Stata. A total of 132 participants were included in the analysis. Results: The respondents were largely females (89%), 71% had a Bachelor’s degree or higher level of education, 90% had some form of employment and 93% were non-smokers. More than half the respondents reported being unaware of Australian dietary fat recommendations. Only 7% agreed that they followed these recommendations, while 37% reported the guidelines were difficult to access and 24% expressed uncertainty about their accessibility. Although most participants considered saturated fats as detrimental and omega-3 polyunsaturated fats as beneficial for health, awareness and adherence to national dietary fat recommendations remained low. Social media was the most frequently reported source of nutrition information (20%), exceeding healthcare professionals (14%). Conclusions: Overall, this survey demonstrates low self-reported awareness and adherence to national dietary fat recommendations and a greater reliance on social media than health professionals for sourcing nutrition information in our study sample. Strategies to improve the accessibility, visibility and communication of dietary fat recommendations may enhance public understanding and may support dietary behaviours beneficial for good overall and cardiovascular health.

## 1. Introduction

Cardiovascular disease (CVD) remains one of the leading causes of morbidity and mortality in Australia [1]. Dietary fats are one of the modifiable risk factors for reducing the risk of CVD [2,3]. Therefore, national dietary guidelines place considerable emphasis on both the quantity and quality of dietary fat consumed as one of the strategies for maintaining or improving cardiovascular health [4]. In Australia, the National Health and Medical Research Council provides the Nutrient Reference Values (NRVs) for dietary fats, along with recommended dietary intakes, and Australian Dietary Guidelines (ADG) to inform the public about the amount and types of foods they should consume [4]. The recommendations are to lower the consumption of total fat and saturated fat to <30% and <10% of total energy intake, respectively [4]. The NRVs are supported by several meta-analyses of randomised trials and observational studies that have linked high intakes of fat, especially saturated fatty acids (SFAs), to adverse cardiometabolic outcomes [2,5]. The NRVs and ADG also recommend substitution of SFAs with polyunsaturated fatty acids (PUFAs) in the diet, while also recommending a lower dietary omega-6 to omega-3 PUFA ratio [4].

Despite the large body of evidence supporting the health-related benefits of NRVs and ADG around dietary fats [6,7], there is some evidence to suggest that Australian adults do [2,3] not consistently adhere to these dietary recommendations [8]. For example, the 2023 National Nutrition and Physical Activity Survey found that Australian adults consume approximately 12.5% of total energy from SFAs, exceeding the recommended limit of 10% [9]. Higher SFA intake may have implications for cardiovascular health, particularly when considered within the context of the overall dietary pattern. Evidence shows that replacing just 5% of energy from SFAs with PUFAs is associated with a 25% lower risk of coronary heart disease [7]. Thus, dietary behaviours not aligning with NRVs and ADG may impact diet quality and may potentially increase the risk associated with cardiometabolic diseases in Australians [10].

One of the key contributors to poor adherence to dietary guidelines can be limited awareness regarding both the quantity and quality of dietary fats required to support optimal health and may reduce cardiometabolic disease risk, as well as a lack of understanding of the food sources that provide different types of fats [11]. Furthermore, dietary fat recommendations have evolved from a focus on reducing total fat intake to emphasising the consumption of healthier fat types, which may contribute to public confusion and inconsistent dietary practices [12]. Despite the importance of dietary fat recommendations for cardiovascular and overall health, contemporary evidence describing the awareness, beliefs, and adherence to these recommendations in Australian adults remains limited. Therefore, this cross-sectional online survey was conducted to examine self-reported awareness, beliefs and adherence to dietary fat recommendations in Australian adults aged 18–45 years, as well as to understand their primary sources of nutrition information.

## 2. Materials and Methods

### 2.1. Study Design

This was an exploratory cross-sectional survey. Given its exploratory nature a sample size was not calculated. Recruitment was set at a minimum of 100 survey respondents. The online survey was administered via Qualtrics (Qualtrics, Provo, UT, USA) from October 2023 to November 2023.

### 2.2. Participants

The survey was advertised through social media posts, flyers, and the university website. Interested participants were able to access the survey using a link or a QR code and self-screened for eligibility. Participants were eligible to complete the survey if they were an Australian adult aged 18–45 years, were able to understand English, and had access to a phone or computer to complete the online survey. Individuals were excluded if they were living outside Australia at the time of survey completion. All eligible participants provided informed consent electronically prior to starting the online survey; this involved reading a Plain Language Statement outlining the study aims and procedures and indicating consent by selecting an agreement option before being granted access to the survey. Ethics approval was obtained from the Deakin University Human Ethics Advisory Group (Project No: HEAG-H 136_2023, Approval date: 13 September 2023).

### 2.3. Survey Questions

The survey was first pilot tested by the research team to assess the comprehensibility and readability of the questions. The final survey comprised up to 33 questions, depending on participant responses, with display logic used to skip non-relevant items. Questions were presented using a combination of tick/multiple-choice responses, 5-point Likert scales (e.g., ranging from strongly disagree to strongly agree), and optional open-text responses. The survey collected data across several domains, including (i) sociodemographic, anthropometric and general health characteristics, (ii) physical activity status, and (iii) awareness, beliefs, and practices surrounding dietary fats.

### 2.4. Sociodemographic, Anthropometric and General Health Characteristics

A combination of tick/multiple-choice and open-text responses were used for questions regarding (1) participant characteristics: age (18–25, 26–30, 31–35, 36–40, and 41–45 years), biological sex at birth (male or female), gender (male, female, non-binary/third gender, transgender, and other), ethnicity (Oceanian, Aboriginal or Torres Strait Islander, Western European, South Asian, Southeast Asian, British, Irish, Eastern European, Middle Eastern, Latin American, Caribbean, North American, African, I don’t know/unsure, and other), education level (Year 11 or below, Year 12, Certificate III/IV, Advance Diploma and Diploma, Bachelor’s degree, Graduate Diploma and Graduate Certificate, Honours/Master, and PhD), and employment status (casual, full-time, part-time, unemployed, and other); (2) anthropometric measures: height in feet or cm and weight in kgs, (3) general health information: existing health conditions, smoking status (yes and no), and alcohol consumption (yes occasionally, yes frequently, and no); and (4) and primary sources for staying up-to-date on nutrition or supplementation strategies for improving your health and wellbeing (celebrities, celebrity chefs, social media, news articles, online health blogs, scientific publications/reports/conferences, doctor/GP and other). Body weight and height data were used to calculate body mass index (BMI) calculated as kg/m^2^ and BMI was categorised as underweight, normal weight, overweight, and obesity based on World Health Organisation classification [13].

### 2.5. Physical Activity Status

Open-text responses were used for questions regarding participants’ physical activity level. They were asked, in the past 7 days, how many days they spent walking, doing endurance activity, and doing resistance activity, and the estimated minutes spent doing those activities per day. The total weekly duration of each type of physical activity was calculated by multiplying the time spent (in minutes) by the number of days.

### 2.6. Awareness, Beliefs, and Preferences Surrounding Dietary Fats, Foods Rich in Fats and Supplements Enriched in Fats/Oil

Nine 5-point Likert scale (ranging from strongly disagree to strongly agree) questions were asked to assess participants’ agreement towards awareness, understanding, adherence, accessibility, feasibility, and trusting the exact amount of fat recommended for good health in the NRVs and the specific Eat for Health Australian dietary guidelines on fat consumption set by the Australian Government. Nine 5-point Likert scale (ranging from highly detrimental to highly beneficial) questions assessed participants’ beliefs regarding the health effects of the amount of fat in the diet, different types of fats (saturated, polyunsaturated and monounsaturated) and their health effects. ‘Unsure’ was included as a separate response option and was treated as a distinct categorical response rather than as a point on the 5-point Likert agreement scale. A combination of tick/multiple-choice and open-text responses was used for questions regarding the type and dosage of fat supplements the participants used and the primary goal and reason for taking them.

### 2.7. Statistical Analysis

Survey data were downloaded from Qualtrics as SPSS and Excel files. Data were analysed using Stata (version 18, StataCorp, College Station, TX, USA). The data were visually inspected for normality by plotting a histogram in Stata. Normality for data showing skewness in the histogram was confirmed using the Shapiro–Wilk test. Only physical activity data were identified as not having a normal distribution and these data are presented as mean ± standard deviation (SD) as well as median with interquartile range. Sociodemographic and general health data are reported as *N* (number of responses) and percentage. Body weight and height data are reported as mean ± SD. The data for questions where participants can select a single answer are reported as *N* (number of responses) and percentage. The data for questions where participants can select multiple answers are reported as frequency and percentage (relative frequency). Responses in some categories were combined for Fisher’s exact test. Specifically, for education level, the following categories: Year 11 or below (including Certificate I/II), Year 12, Certificate III/Iv and Advanced Diploma and Diploma, were combined into a single category labelled ‘Below Bachelor’s degree’. For primary sources of information used to stay up-to-date on nutrition or supplementation strategies for health and wellbeing, the categories comprising online health blogs/forums, celebrities, celebrity chefs, and social media platforms (e.g., Instagram and Facebook) were combined into a category called ‘Celebrities/social media/Online Blogs/Forums’. Additionally, news articles (print and digital) and other sources were combined into a category labelled ‘Other Sources/News Articles’. Responses to 5-point Likert scale questions (ranging from strongly disagree to strongly agree) were combined into four categories: Agree, Disagree, Neutral, and Unsure. Similarly, responses to 5-point Likert scale questions (ranging from highly detrimental to highly beneficial) were grouped into Beneficial, Detrimental, Neutral, and Unsure categories. Significant associations between participant characteristics (employment status, age group, BMI category, primary sources of nutrition/supplementation information, employment status and education level) and participants’ awareness, beliefs and practices regarding dietary fats were assessed using Fisher’s exact test. Since these analyses were exploratory and hypothesis-generating rather than confirmatory, no adjustment for multiple testing was applied [14]. Statistical significance was set at *p* < 0.05. Post hoc estimation using adjusted standardised residuals was not performed due to small sample size (*N* < 5) in some response categories.

## 3. Results

### 3.1. Survey Response

A total of 145 respondents completed the survey. Of these, three were identified as having an IP address of an international country and were excluded. As per Qualtrics fraud detection protocol, the responses that had a relevant ID duplicate score of >75 (*N* = 6) or had a relevant ID fraud score of >30 (*N* = 1) were excluded. Three people completed <10% of the survey and were excluded. The remaining 132 responses were included in the analysis. The final sample size varied by question, as not all respondents answered all questions, the sample size for each question has been reported individually.

### 3.2. Participant Characteristics

Participant characteristics are given in Table 1. Of those who completed the survey, 89% were biologically female and 11% were biologically male. The mean ± SD self-reported weight was 70 ± 20 kg, and the mean height was 166 ± 8 cm. Participants were distributed across age groups, with the largest proportions aged 41–45 years (22%) and 26–30 years (22%), followed by 18–25 years (21%). Oceanian was the most common ethnicity (42%), followed by Southeast Asian (22%) and British (16%). Educational attainment varied, with 30% holding an Honours or Master’s degree, 23% a Bachelor’s degree, and 8% a PhD. Employment status was predominantly full-time (46%), with 21% employed casually, 15% part-time and 10% unemployed. The majority of the participants (93%) were non-smokers, 56% occasional alcohol consumers, 6% frequent alcohol consumers and 37% reported they did not consume alcohol.

As shown in Table 2, participants also reported that they engaged in walking on average (mean ± SD) 5 ± 2 days per week (for 42 ± 49 min per day), in endurance activity on 2 ± 2 days per week (for 25 ± 34 min per day), and in resistance activity on 1 ± 2 days per week (15 ± 24 min per day).

### 3.3. Participants’ Awareness of NRVs and ADG

Participants’ self-reported awareness of NRVs and Eat for Health ADG are presented in Table 3. The majority of participants disagreed (36% strongly disagreed, 17% somewhat disagreed) with the statement that they were aware of the exact amount of fat recommended for good health in NRVs; many were unsure (21%) and only 12% somewhat or strongly agreed. A similar pattern was seen for NRVs’ adherence where 48% of the participants somewhat or strongly disagreed that they always follow the exact amount of fat recommended for good health in NRVs, 24% were unsure and only 7% somewhat or strongly agreed. When asked about the Eat for Health ADG specifically, 57% of participants somewhat or strongly disagreed with the statement “I am aware of the specific Eat for Health ADG on fat consumption outlined by the Australian Government”, 17% were unsure and only 18% somewhat or strongly agreed. Only 12% of the participants somewhat or strongly agreed that they follow the Eat for Health ADG, 47% somewhat or strongly disagreed and 27% were unsure.

Participants were also asked if they agreed with the NRVs and ADG and found them comprehensible and accessible (Table 3). When asked if NRVs and ADG are easy to access, 37% somewhat or strongly disagreed while one-quarter of participants (24%) reported being unsure and only 23% agreed. When asked if the guidelines were easy to understand, 22% somewhat or strongly disagreed and 32% were unsure and only 28% of participants somewhat or strongly agreed. Despite a larger number of participants not finding the NRVs and ADG accessible and being uncertain about understanding the guidelines, 49% of participants somewhat or strongly agreed that they trust the information provided in the NRVs and the specific Eat for Health ADG, 15% somewhat or strongly disagreed and 19% were unsure.

### 3.4. Participants’ Awareness Around Dietary Fats

Participant responses regarding their self-reported awareness of health effects of different types of fats are presented in Table 4. For the statement “I don’t think a high fat diet is bad for health”, 53% of the participants somewhat or strongly disagreed, 31% somewhat or strongly agreed, and 14% neither agreed nor disagreed. Most of the participants (78%) somewhat or strongly agreed that “a high-fat diet is bad for health, but some fat is okay”. When asked about the statement “I think dietary fat is bad for health in general regardless of amount and type”, 14% somewhat or strongly agreed but 69% somewhat or strongly disagreed. When asked if participants always choose low-fat foods for health reasons, 48% somewhat or strongly disagreed with this statement, 34% somewhat or strongly agreed and 16% remained neutral.

Regarding the participants’ self-reported awareness about the health effects of specific types of fat, the responses varied. For the statement “I am fully aware of the health effects of different types of fats (saturated, polyunsaturated and monounsaturated [MUFA])” 62% of the participants somewhat or strongly agreed, 20% somewhat or strongly disagreed and 13% remained neutral. For omega-6 PUFAs, 59% considered them beneficial, 14% considered them detrimental and 18% were unsure. For omega-3 PUFAs, 75% of participants believed them to be beneficial, 7% believed them to be detrimental and 14% were unsure. For MUFAs, 37% believed them to be beneficial, 16% considered them detrimental and 31% were unsure. For saturated fats, 72% of the participants believed they are detrimental for health while 7% believed them to be beneficial, 9% were neutral and 12% were unsure.

### 3.5. Participants’ Beliefs and Practices Around Use of Supplements Containing Dietary Fats

**Figure 1:**
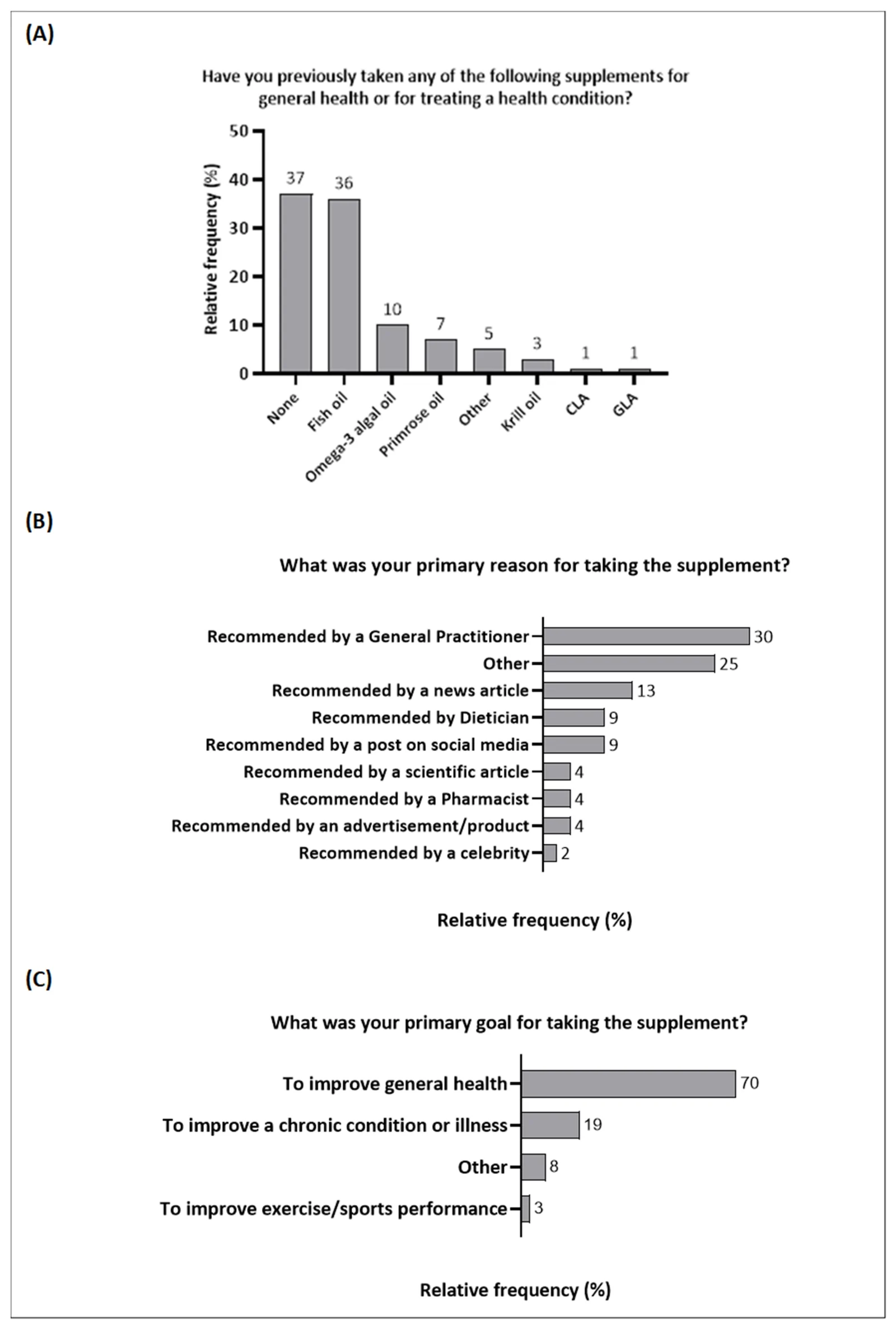
Use of supplements containing dietary fats and oils amongst Australian adults aged 18–45 years. (**A**) Supplements previously used by the participants; (**B**) Primary reason for taking the supplement (**C**) Primary goal for taking the supplement.

The responses of the participants who answered questions regarding their use of fat-based supplements are presented in Figure 1, which outlines the types of supplements previously taken (Figure 1A), the primary reasons for their use (Figure 1B), and the main health goals participants aimed to achieve through taking the supplements (Figure 1C). When participants were asked whether they had previously taken supplements containing dietary fats, the most common response was taking none (37%). Among those who had taken supplements, fish oil was by far the most commonly reported option (36%), followed by omega-3 algal oil (10%) and primrose oil (7%). Among those who had taken a supplement, the most frequently reported primary reason for doing so was a recommendation from a General Practitioner (GP) (30%), followed by the option ‘other’ (25%). The responses given by the participants for ‘other’ mainly included recommended by parent or partner. Regarding participants’ primary goals for using fat-based supplements, the majority indicated they took them to improve general health (70%) with the second most common goal being to improve a chronic condition or illness (19%).

**Figure 2:**
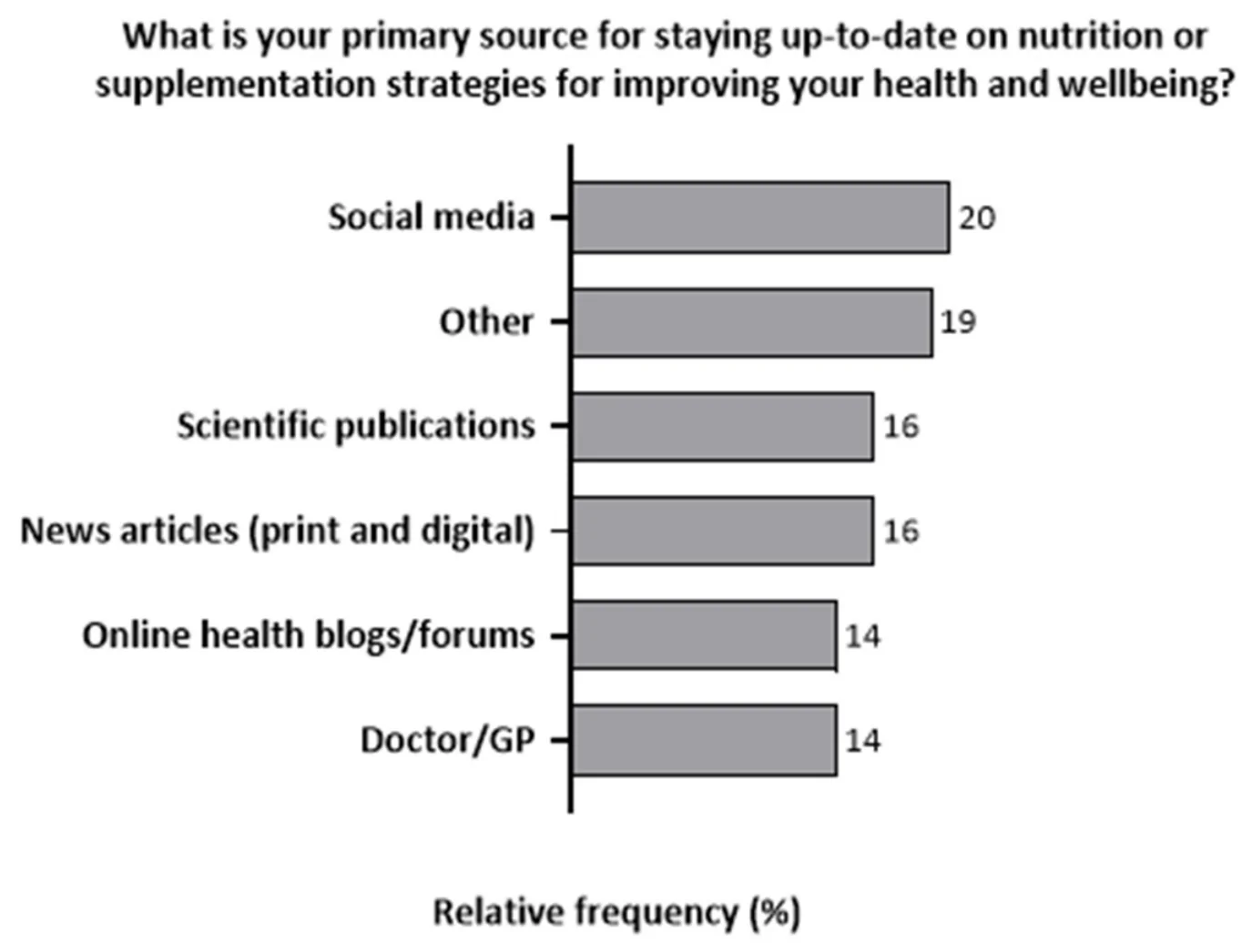
Nutrition information sources used by Australian adults aged 18–45 years.

**Figure 3:**
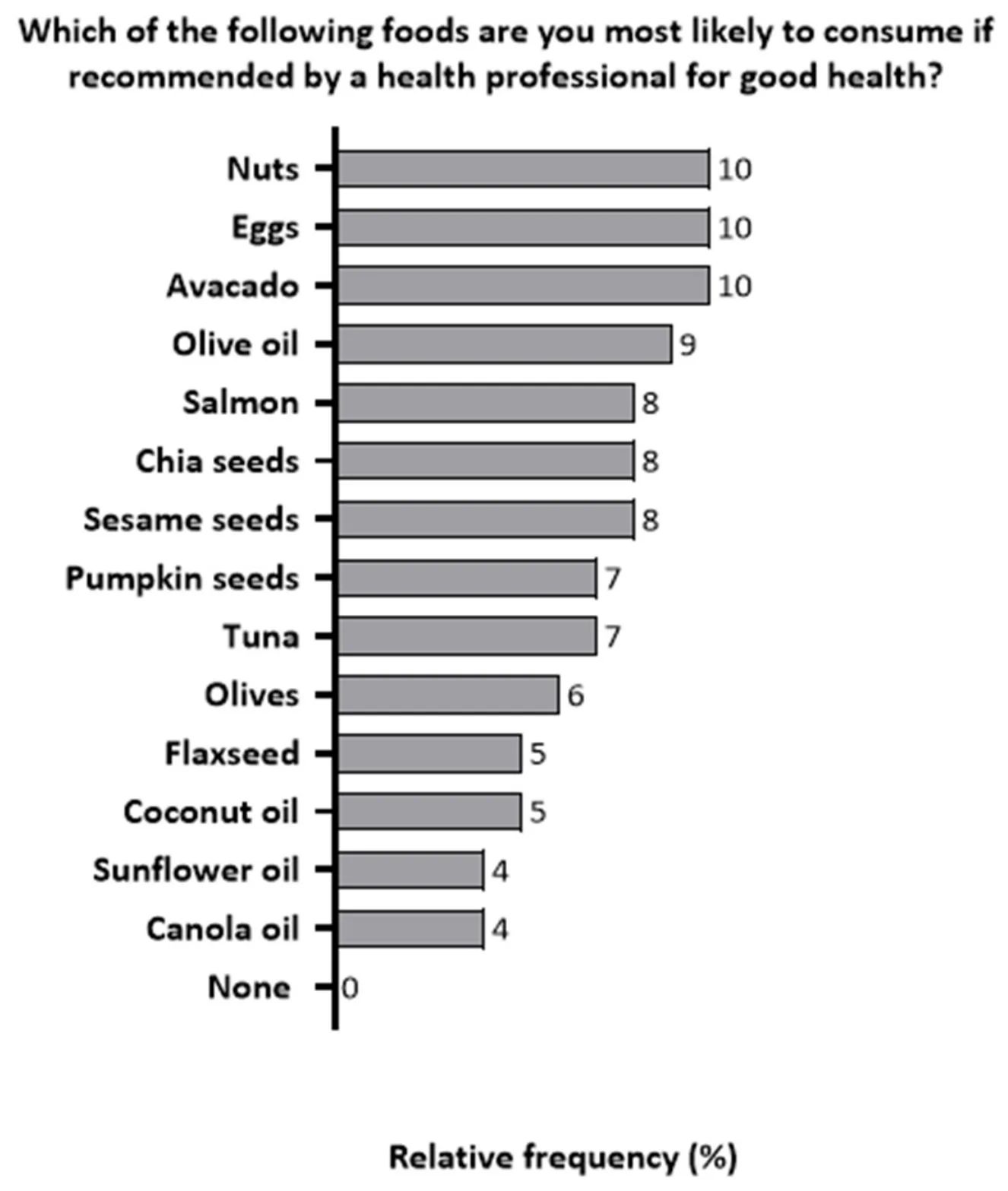
Dietary fat rich foods preferred by Australian adults aged 18–45 years.

When participants were asked “what was their primary source for staying up-to-date on nutrition or supplementation strategies for improving health and well-being” (Figure 2), the most common response was social media (20%) followed by other (19%). Under the ‘other’ option, participants’ responses included dietitian (*N* = 8), nutritionist (*N* = 2), family and friends (*N* = 2). GP was the least reported option (14%) to use as a primary source for staying up-to-date on nutrition or supplementation strategies. When asked which of the following foods the participants were most likely to consume if recommended by a health professional (Figure 3), nuts, eggs, and avocado were the most popular choices (10% each) followed by olive oil (9%), salmon (8%), chia seeds (8%), sesame seeds (8%), pumpkin seeds (7%), and tuna (7%).

### 3.6. Associations Between Participant Characteristics and Their Awareness, Beliefs and Practices Around Dietary Fats

The associations between participant characteristics (age, BMI, education and employment status) and their awareness, beliefs and practices around dietary fats are provided in Table 5, with significant associations between participant characteristics and responses in Supplementary Table S1. The results show that BMI showed no significant association with participants’ awareness, beliefs and practices around dietary fats. However, age showed a significant association with the statement that “I think Nutrient Reference Values and Australian dietary guidelines are easy to access” (*p* = 0.017).

However post hoc estimation using adjusted standardised residuals was not performed due to small sample size (*N* < 5) in some response categories; therefore, the direction of the associations remains unclear. Employment status demonstrated a significant association with the statements that “I am aware of the exact amount of fat recommended for good health in the Nutrient Reference Values set by the Australian Government” (*p* = 0.025) and “I always follow the exact amount of fat recommended for good health in the Nutrient Reference Values set by the Australian Government” (*p* = 0.034); however, the direction of this association was unclear (Supplementary Table S1). Education status showed a significant association with participants’ belief that “I think a high fat diet is bad for health, but some fat is okay” (*p* = 0.039); however, in the absence of post hoc analysis, the direction of the associations remains unclear.

### 3.7. Associations Between Participants’ Nutrition Information Source and Their Current Awareness, Beliefs and Practices Around Dietary Fats

The associations between participants’ nutrition information sources (celebrities/social media/online blogs/forums; doctor/general practitioners/pharmacist; other sources/news articles; scientific publications/reports/and conferences) and their awareness, beliefs and practices around dietary fats are presented in Table 6.

A detailed response data set for these significant associations has been presented in Supplementary Table S2. A significant association was observed (*p* = 0.016) between participants’ information source and participants’ practices that “I always follow the exact amount of fat recommended for good health in the Nutrient Reference Values set by the Australian Government”. A significant association (*p* = 0.009) was observed between participants’ information source and participants’ belief on “How do you think saturated fat is likely to affect your health?”, however the direction of this association was unclear (Supplementary Table S2). No other significant associations were observed between participants’ information sources and the remaining questions regarding awareness, belief and practice statements related to dietary fat.

## 4. Discussion

This study investigated self-reported awareness, beliefs, and adherence to national recommendations on dietary fat in Australian adults aged between 18 and 45 years. The findings revealed low awareness, understanding, and adherence to the NRVs and ADG, despite the central role these recommendations play in promoting healthy dietary patterns. Although most participants identified SFAs as detrimental and omega-3 PUFAs as beneficial, substantial uncertainty remained regarding the broader application of dietary fat guidance and the health effects of specific fat types. Collectively, these findings highlight a disconnect between dietary recommendations and consumer awareness and behaviour in relation to dietary fats.

The findings showed that self-reported awareness around dietary fat recommendations in NRVs and ADG is generally low among Australian adults aged between 18 and 45 years with only 12% of participants reporting being aware of them. Many participants (21%) also reported being unsure which suggests a lack of awareness and/or understanding around guidelines on dietary fats. Consistent with the finding of limited awareness, participants also reported low adherence (only 7% agreed they follow the guidelines). Similar results have been observed in Dutch and American populations where a lack of awareness about one’s fat intake contributed to a lack of adherence [15]. These findings suggest that the translation of the dietary fat recommendations into population-level dietary behaviours remains suboptimal. This implementation gap may partly explain why Australians continue to exceed the recommended saturated fat intakes [9], despite longstanding public health messaging.

In our study, participants also reported difficulties related to accessibility and comprehensibility of the dietary guidelines which may be a contributing factor to lower awareness and adherence to these recommendations. A substantial proportion of participants either disagreed that the guidelines are easy to access (37%) and understand (22%) or were simply unsure (24–32%). Consistent with our findings, previous evidence has shown that recommendations related to fat intake are among the least well understood and least applied components of the Dietary Guidelines in the American population [16].

While most participants recognised saturated fats as being detrimental and omega-6 and omega-3 fats as beneficial, there was considerable uncertainty around the health effects of MUFAs (with 31% reported being unsure). This may be due to long-standing public health messaging from major organisations that has strongly emphasised the benefits of omega-3 fats and the detrimental effects of saturated fats for cardiovascular health, with comparatively less emphasis on other fat types [4,17]. Greater public understanding of the differences between saturated, monounsaturated, and polyunsaturated fats may support healthier food choices and may promote better adherence to cardioprotective dietary patterns.

Another notable finding was the influence of participant characteristics and information sources on dietary fat-related awareness and practices. The findings of the present study suggest that beliefs, awareness and practices related to dietary fat may be influenced by participant characteristics, age, education and employment status, as well as by sources of nutrition information. Although the direction of these associations could not be determined because of the limited sample size, the findings support the need for targeted communication strategies that account for demographic differences in health literacy and information seeking behaviour. Tailored approaches may be particularly important for reaching groups that are less engaged with traditional health communication channels and may therefore be at greater risk of misunderstanding dietary guidance relevant to cardiovascular health [18].

We also surveyed the use of supplements containing dietary fats and oils in our study and found omega-3 supplements in the form of fish oil as being the most reported supplement consumed by this cohort. Participants reported that the main goal for using dietary fat supplements was to improve general health. Similar findings have been reported by a study analysing the 2007–2010 NHANES survey in the United States [19]. They found that after multivitamin and calcium supplements, omega-3 supplements were the third most commonly used supplement amongst the 11,956 respondents. This study also reported that the most common reasons that adults reported using dietary supplements were to “improve overall health” and to “maintain health”, which aligns with the findings of our study [19]. Given that current dietary guidelines prioritise obtaining nutrients from whole foods and dietary patterns where possible, future research should explore whether improving public understanding of food-based sources of healthy fats could reduce reliance on supplementation and encourage greater adherence to dietary recommendations.

In our study, among individuals who reported supplement use, recommendations from a GP were the most cited primary reason. Even though participants reported consuming supplements due to GP recommendations, social media emerged as the most frequently reported source of nutrition information, while healthcare professionals were less commonly cited. Furthermore, the nutrition information source was associated with both perceptions of saturated fat and self-reported adherence to dietary recommendations. These findings suggest a potential influence of social media on beliefs and behaviours related to dietary fats. A previous study evaluating participants’ diet-information seeking behaviour reported that the top three most sought-after sources were ‘nutrition or health websites’, ‘Google or internet search’ and ‘diet or health books’. Social media was ranked number eight while GPs ranked number seventeen [20]. Use of the internet and social media has been linked to negative body image, unhealthy dietary trends and disordered eating [21,22]. Our results suggest that there is a growing need for health professionals, researchers, and public health agencies to establish a stronger presence across digital media platforms and provide evidence-based, accessible content that translates complex nutrition recommendations into practical dietary advice.

It is important to acknowledge that dietary fat intake is only one component affecting cardiovascular health and interacts with other behaviours such as physical activity, smoking, alcohol consumption, sleep, stress and weight management [23]. Participants in the current study generally reported favourable lifestyle behaviours, including low smoking prevalence and engagement in physical activity, yet awareness and adherence to dietary fat guidance remained low. This suggests that nutrition literacy may represent a distinct target for intervention, even among individuals who engage in other health-promoting behaviours. Future studies should explore how dietary awareness interacts with broader lifestyle behaviours to influence long-term health outcomes.

Our survey provides novel insights into self-reported awareness beliefs and adherence to dietary fat recommendations amongst Australian adults aged between 18 and 45 years. The inclusion of questions on information sources and supplement use provides additional context to understand behavioural drivers. Furthermore, the exploratory design allows for identification of emerging trends and hypothesis generation for future research. However, there are several limitations that need to be considered. Our sample size is small and overrepresented by females, and individuals with higher educational attainment. Furthermore, recruitment primarily through social media may have introduced selection bias by preferentially reaching individuals who were more engaged with online platforms, which reduces the generalisability to the entire adult Australian population. Moreover, household or individual income was not assessed, limiting our ability to examine the potential influence of socioeconomic circumstances on awareness, accessibility, and adherence to dietary fat recommendations. The survey questionnaire was not validated; however, it was pilot tested to improve participant comprehension. Finally, the self-reported nature of the survey may introduce some bias, as dietary behaviours may differ from the responses [24].

## 5. Conclusions

Overall, the findings suggest that improving dietary fat literacy may represent an important and scalable strategy for improving adherence to dietary guidelines amongst Australians aged 18–45 years. While public trust in dietary guidelines appears reasonably strong, awareness, accessibility, and practical application remain limited. Bridging this gap will require communication strategies that prioritise food-based dietary patterns, leverage digital communication platforms, and tailor messages to the needs of diverse population groups. Such approaches may enhance adherence to dietary recommendations and ultimately may contribute to dietary behaviours beneficial for good overall and cardiovascular health.

## Figures and Tables

**Table 1:** Participant characteristics.

Participant Characteristics	*N* or Frequency Unless Otherwise Stated	Percentage (%)
Biological sex		
Male	12	10.8
Female	99	89.2
Self-identified gender		
Male	12	10.8
Female	93	83.8
Non-binary/third gender	4	3.6
Transgender	1	0.9
Other	1	0.9
Total respondents	111	100
Self-reported weight (Mean ± SD)	70 ± 20	
Total respondents	102	
Self-reported height (Mean ± SD)	166 ± 8	
Total respondents	99	
Self-reported age (in years)		
18–25	23	20.7
26–30	24	21.6
31–35	17	15.3
36–40	22	19.8
41–45	25	22.5
Total respondents	111	100
Ethnicity		
Aboriginal or Torres Strait Islander	1	0.9
African	2	1.8
British	18	16.2
Eastern European	8	7.2
Irish	6	5.4
Latin American	5	4.5
Middle Eastern	4	3.6
North American	4	3.6
Oceanian	47	42.3
South Asian	8	7.2
Southeast Asian	24	21.6
Western European	6	5.4
Other	5	4.5
Total respondents	111	100
Highest education		
Year 11 or below	2	1.8
Year 12	14	12.6
Advanced Diploma and Diploma	8	7.2
Bachelor’s degree	26	23.4
Certificate III/IV	7	6.3
Doctor of Philosophy	9	8.1
Graduate Diploma and Graduate Certificate	11	9.9
Honours and/or master’s degree	33	29.7
Other	1	0.9
Total respondents	111	100
Employment status		
Casual	23	20.7
Full-time	51	46.0
Unemployed	11	9.9
Part-time	17	15.3
Other	9	8.1
Total respondents	111	100
Smokers		
Yes	9	6.9
No	122	93.1
Total respondents	131	100
Alcohol		
Yes occasionally	74	56.4
Yes frequently	8	6.1
No	49	37.4
Total respondents	131	100

**Table 2:** Self-reported type and duration of physical activity.

Type of Physical Activity	Number of Days per Week	Minutes Spent per Day
Walking (Mean ± SD)	5 ± 2	42 ± 49
Median (IQR)	6 (4, 7)	30 (20, 50)
Endurance physical activity (Mean ± SD)	2 ± 2	24 ± 34
Median (IQR)	1 (0, 3)	13.5 (0, 45)
Resistance physical activity (Mean ± SD)	1 ± 2	15 ± 24
Median (IQR)	0 (0, 2)	0 (0, 25)
Total respondents	111	

**Table 3:** Self-reported awareness and adherence to Nutrient Reference Values (NRVs) and Eat for Health Australian dietary guidelines.

Question	Response	*N* (%)
I am aware of the exact amount of fat recommended for good health in NRVs set by the Australian Government	Somewhat agree	10 (8.3)
	Strongly agree	5 (4.2)
	Neither agree nor disagree	17 (14.2)
	Somewhat disagree	20 (16.7)
	Strongly disagree	43 (35.8)
	Unsure	25 (20.8)
	Total respondents	120 (100.0)
I always follow the exact amount of fat recommended for good health in NRVs set by Australian Government	Somewhat agree	5 (4.1)
	Strongly agree	3 (2.5)
	Neither agree nor disagree	25 (20.8)
	Somewhat disagree	18 (15.0)
	Strongly disagree	40 (33.3)
	Unsure	29 (24.2)
	Total respondents	120 (100.0)
I am aware of the specific Eat For Health Australian dietary guidelines on fat consumption outlined by Australian Government	Somewhat agree	16 (13.3)
	Strongly agree	6 (5.0)
	Neither agree nor disagree	10 (8.3)
	Somewhat disagree	23 (19.2)
	Strongly disagree	45 (37.5)
	Unsure	20 (16.7)
	Total respondents	120 (100.0)
I always follow the specific Eat For Health Australian dietary guidelines on fat consumption outlined by Australian Government	Somewhat agree	11 (9.2)
	Strongly agree	4 (3.3)
	Neither agree nor disagree	17 (14.2)
	Somewhat disagree	19 (15.8)
	Strongly disagree	37 (30.8)
	Unsure	32 (26.7)
	Total respondents	120 (100.0)
I think Nutrient Reference Values (NRVs) and Australian dietary guidelines are easy to access	Somewhat agree	19 (15.8)
	Strongly agree	9 (7.5)
	Neither agree nor disagree	19 (15.8)
	Somewhat disagree	21 (17.5)
	Strongly disagree	23 (19.2)
	Unsure	29 (24.2)
	Total respondents	120 (100.0)
I think Nutrient Reference Values (NRVs) and Australian dietary guidelines are easy to understand	Somewhat agree	27 (22.5)
	Strongly agree	7 (5.8)
	Neither agree nor disagree	21 (17.5)
	Somewhat disagree	13 (10.8)
	Strongly disagree	13 (10.8)
	Unsure	39 (32.5)
	Total respondents	120 (100.0)
I trust the information provided in Nutrient Reference Values (NRVs) and Australian dietary guidelines	Somewhat agree	46 (38.3)
	Strongly agree	13 (10.8)
	Neither agree nor disagree	20 (16.7)
	Somewhat disagree	12 (10.0)
	Strongly disagree	6 (5.0)
	Unsure	23 (19.2)
	Total respondents	120 (100.0)

**Table 4:** Participants’ awareness of health effects of dietary fats.

Question	Response	*N* (%)
I don’t think a high fat diet is bad for health	Somewhat agree	29 (24.2)
	Strongly agree	8 (6.7)
	Neither agree nor disagree	17 (14.2)
	Somewhat disagree	31 (25.8)
	Strongly disagree	33 (27.5)
	Unsure	2 (1.7)
	Total respondents	120 (100.0)
I think a high fat diet is bad for health but some fat is okay	Somewhat agree	48 (40.3)
	Strongly agree	45 (37.8)
	Neither agree nor disagree	10 (8.4)
	Somewhat disagree	7 (5.9)
	Strongly disagree	8 (6.7)
	Unsure	1 (0.8)
	Total respondents	119 (100.0)
I think dietary fat is bad for health in general regardless of amount and type	Somewhat agree	12 (10.1)
	Strongly agree	4 (3.6)
	Neither agree nor disagree	16 (13.4)
	Somewhat disagree	25 (21.0)
	Strongly disagree	57 (47.9)
	Unsure	5 (4.2)
	Total respondents	119 (100.0)
I always choose low fat foods over high fat foods for health reasons	Somewhat agree	28 (23.5)
	Strongly agree	13 (10.9)
	Neither agree nor disagree	19 (16.0)
	Somewhat disagree	30 (25.2)
	Strongly disagree	27 (22.7)
	Unsure	2 (1.7)
	Total respondents	119 (100.0)
I am fully aware of the health effects of different types of fats (saturated, polyunsaturated and monounsaturated)	Somewhat agree	53 (44.5)
	Strongly agree	21 (17.6)
	Neither agree nor disagree	15 (12.6)
	Somewhat disagree	14 (11.8)
	Strongly disagree	10 (8.4)
	Unsure	6 (5.0)
	Total respondents	119 (100.0)
How do you think omega-6 polyunsaturated fats are likely to affect your health?	Highly beneficial	29 (24.2)
	Somewhat beneficial	42 (35.0)
	Neither detrimental nor beneficial	10 (8.3)
	Highly detrimental	6 (5.0)
	Somewhat detrimental	11 (9.2)
	Unsure	22 (18.3)
	Total respondents	120 (100.0)
How do you think omega-3 polyunsaturated fats are likely to affect your health?	Highly beneficial	51 (42.5)
	Somewhat beneficial	39 (32.5)
	Neither detrimental nor beneficial	5 (4.2)
	Highly detrimental	3 (2.5)
	Somewhat detrimental	5 (4.2)
	Unsure	17 (14.2)
	Total respondents	120 (100.0)
How do you think monounsaturated fat is likely to affect your health?	Highly beneficial	17 (14.2)
	Somewhat beneficial	27 (22.5)
	Neither detrimental nor beneficial	20 (16.7)
	Highly detrimental	7 (5.8)
	Somewhat detrimental	12 (10.0)
	Unsure	37 (30.8)
	Total respondents	120 (100.0)
How do you think saturated fat is likely to affect your health?	Highly beneficial	4 (3.3)
	Somewhat beneficial	5 (4.2)
	Neither detrimental nor beneficial	11 (9.2)
	Highly detrimental	50 (41.7)
	Somewhat detrimental	36 (30.0)
	Unsure	14 (11.7)
	Total respondents	120 (100.0)

**Table 5:** Association between participant characteristics and their awareness, beliefs and practices around dietary fats.

	Participant Characteristics			
	Age	BMI	Education Status	Employment Status
Statements around dietary fat				
I am fully aware of the health effects of different types of fats (saturated, polyunsaturated and monounsaturated).	0.110	0.167	0.625	0.560
I am aware of the exact amount of fat recommended for good health in Nutrient Reference Value set by the Australian Government.	0.065	0.244	0.528	0.025 *
I always follow the exact amount of fat recommended for good health in the Nutrient Reference Value set by the Australian Government.	0.952	0.097	0.197	0.034 *
I am aware of the specific Eat For Health Australian dietary guidelines on fat consumption outlined by Australian Government.	0.298	0.779	0.975	0.394
I always follow the specific Eat for Health Australian dietary guidelines on fat consumption outlined by Australian Government.	0.881	0.425	0.848	0.394
How do you think omega-3 polyunsaturated fats are likely to affect your health?	0.413	0.594	0.735	0.266
How do you think saturated fat is likely to affect your health?	0.324	0.224	0.845	0.206
I always choose low fat foods over high fat foods for health reasons.	0.107	0.614	0.781	0.674
I don’t think a high fat diet is bad for health.	0.837	0.697	0.563	0.377
I think a high fat diet is bad for health but some fat is okay.	0.085	0.788	0.039 *	0.659
I think dietary fat is bad for health in general regardless of amount and type.	0.911	0.471	0.157	0.194
I think Nutrient Reference Values and Australian dietary guidelines are easy to access.	0.017 *	0.827	0.064	0.570

Data presented is Fisher’s exact test *p*-values. * Denotes significant *p* < 0.05. Post hoc estimation using adjusted standardised residuals was not performed due to small sample size (*N* < 5) in some response categories.

**Table 6:** Association between participants’ nutrition information source and their awareness, beliefs and practices around dietary fats.

	Information Source
Statements around dietary fat	
I am fully aware of the health effects of different types of fats (saturated, polyunsaturated and monounsaturated).	0.545
I am aware of the exact amount of fat recommended for good health in Nutrient Reference Values set by the Australian Government.	0.117
I always follow the exact amount of fat recommended for good health in the Nutrient Reference Values set by the Australian Government.	0.016 *
I am aware of the specific Eat For Health Australian dietary guidelines on fat consumption outlined by Australian Government.	0.089
I always follow the specific Eat for Health Australian dietary guidelines on fat consumption outlined by Australian Government.	0.457
How do you think omega-3 polyunsaturated fats are likely to affect your health?	0.355
How do you think saturated fat is likely to affect your health?	0.009 *
I always choose low fat foods over high fat foods for health reasons.	0.813
I don’t think a high fat diet is bad for health.	0.707
I think a high fat diet is bad for health but some fat is okay.	0.629
I think dietary fat is bad for health in general regardless of amount and type.	0.895
I think Nutrient Reference Values and Australian dietary guidelines are easy to access.	0.388

Data presented is Fisher’s exact test *p*-values. * Denotes significant *p* < 0.05. Nutrition information source options in the survey included—celebrities/social media/online blogs/forums; doctor/general practitioners/pharmacist; other sources/news articles; scientific publications/reports/and conferences. Post hoc estimation using adjusted standardised residuals was not performed due to small sample size (*N* < 5) in some response categories.

## Data Availability

Data is available upon reasonable request to the corresponding author.

## Supplementary Materials

The following supporting information can be downloaded at https://www.mdpi.com/article/10.3390/nu18193236/s1, Supplementary Table S1: Full response data on significant association between participant characteristics and awareness/belief/practices around dietary fat, Supplementary Table S2: Full response data on significant association between participant information source and participants’ awareness/belief/practices around dietary fat.

## Abbreviations

The following abbreviations are used in this manuscript:

ADG	Australian dietary guidelines
NRVs	Nutrient reference values
BMI	Body mass index
SFAs	Saturated fatty acids
PUFAs	Polyunsaturated fatty acids
MUFAs	Monounsaturated fatty acids
