# Supplementary material for: Self-Reported Awareness and Adherence to Recommendations for Dietary Fat Amongst Australian Adults Aged 18–45 Years: Potential Relevance to Cardiovascular Health
Source: Nutrients. 2026 Sep 30;18(19):3236. doi: 10.3390/nu18193236 (PMC13666735; doi:10.3390/nu18193236)
Supplement: Supplementary file 1 [file nutrients-18-03236-s001.zip › nutrients-4561987-supplementary.pdf]

Supplementary Table S1: Full response data on significant association between participant characteristics and awareness/belief/practices around dietary fat

Age (years)								
**Question**	**Response**	**18-25**	**26-30**	**31-35**	**36-40**	**41-45**	**Total**	**p-value**
I think Nutrient Reference Values and Australian dietary guidelines are easy to access.	Unsure	3	3	10	7	5	28	**0.017**
	Agree	10	7	3	1	4	25	
	Disagree	8	8	3	10	11	40	
	Neutral	2	6	1	4	5	18	
	Total	23	24	17	22	25	111	
**Employment status**								
**Question**	**Response**	**Casual**	**Full-time**	**Other**	**Part-time**	**Unemployed**	**Total**	**p-value**
I am aware of the exact amount of fat recommended for good health in Nutrient Reference Values set by the Australian Government.	Unsure	3	7	2	5	6	23	**0.025**
	Agree	1	9	1	1	2	14	
	Disagree	16	29	3	10	1	59	
	Neutral	3	6	3	1	2	15	
	Total	23	51	9	17	11	111	

Employment status								
**Question**	**Response**	**Casual**	**Full-time**	**Other**	**Part-time**	**Unemployed**	**Total**	**p-value**
I always follow the exact amount of fat recommended for good health in the Nutrient Reference Values set by the Australian Government.	Unsure	5	14	1	3	5	28	**0.034**
	Agree	1	5	0	0	1	7	
	Disagree	14	20	4	14	2	54	
	Neutral	3	12	4	0	3	22	
	Total	23	51	9	17	11	111	

Education									
**Question**	**Response**	**Graduate Diploma and Diploma certificate**	**Bachelor's degree**	**Doctor of Philosophy**	**Honours and/or Master's Degree**	**Other**	**Under Bachelor's degree**	**Total**	**p-value**
I think a high fat diet is bad for health but some fat is okay.	Unsure	0	0	0	1	0	0	1	**0.039**
	Agree	7	19	7	31	1	23	88	
	Disagree	4	3	2	0	0	3	12	

	Neutral	0	4	0	1	0	4	9	
	Total	11	26	9	33	1	30	110	

Data are presented as number of responses (n) in each category.

Supplementary Table S2: Full response data on significant association between participant information source and participants' awareness/belief/practices around dietary fat.

**Information source**							
**Question**	**Response**	**Celebrities/ social media/ Online Blogs/ Forums**	**Doctor/GP/ Pharmacist**	**Other Sources/ News Articles**	**Scientific publications, reports, and conferences**	**Total**	**p-value**
I always follow the exact amount of fat recommended for good health in the	Unsure	12	6	9	2	29	**0.016**
	Agree	1	5	2	0	8	
	Disagree	20	6	21	11	58	

Nutrient Reference Values set by the Australian Government.	Neutral	11	0	9	5	25	
	Total	44	17	41	18	120	
**Information source**							
**Question**	**Response**	**Celebrities/ social media/ Online Blogs/ Forums**	**Doctor/GP/ Pharmacist**	**Other Sources/ News Articles**	**Scientific publications, reports, and conferences**	**Total**	**p-value**
How do you think saturated fat is likely to affect your health?	Unsure	8	3	3	0	14	**0.009**
	Beneficial	4	3	1	1	9	
	Detrimental	30	10	35	11	86	
	Neutral	2	1	2	6	11	
	Total	44	17	41	18	120	

Data are presented as number of responses in each category.
